# Impact of Malocclusions on the Oral Health-Related Quality of Life of Early Adolescents in Ndola, Zambia

**DOI:** 10.1155/2018/7920973

**Published:** 2018-06-03

**Authors:** Severine N. Anthony, Kayembe Zimba, Balakrishnan Subramanian

**Affiliations:** ^1^Department of Dental Clinical Sciences, Michael Chilufya Sata School of Medicine, Copperbelt University, Ndola, Zambia; ^2^Department of Basic Medical Sciences, Michael Chilufya Sata School of Medicine, Copperbelt University, Ndola, Zambia

## Abstract

The study aimed to assess the prevalence of malocclusions and its impact on oral health-related quality of life (OHRQoL) among early adolescents in Ndola, Zambia. It used a random sample of 384 primary school children aged 12–14 years. The Child Oral Health Impact Profile-Short Form 19 (COHIP-SF19) was used to assess OHRQoL, and the Dental Aesthetic Index (DAI) was used to examine dentofacial anomalies. The chi-square test was used to study whether there was a statistically significant association between variables and multivariate logistic regression for the influence of sociodemographic and malocclusions on OHRQoL. Statistical significance was set at *p* < 0.05. Participants' sociodemographics were 53.6% female, 41.7% aged 13 years, and 43.5% from grade six. The overall reported impact on OHRQoL was 11.7%, which was significant (*p* < 0.001) by age and sex, and higher in females than males. The overall prevalence of malocclusions was 27.9%, which was significant (*p*=0.005) by sex, and higher in males than females. Children with malocclusions reported significant (*p* < 0.001) negative oral health impact compared to the children without malocclusions. Spacing, diastema, and crowding were most prevalent malocclusions that showed clear inverse association with OHRQoL. The study findings provide indications that malocclusions are negatively associated with OHRQoL among Zambian early adolescents.

## 1. Introduction

The impact of oral health on one's quality of life is termed as oral health-related quality of life (OHRQoL), which is used to assess how pain/discomfort and physical, psychological, and social functions affect well-being [1]. OHRQoL is associated with self-related oral health; subjective symptoms of temporomandibular disorders (TMDs); oral pain and stomatitis; decayed, missing, and filled teeth (DMFT); and malocclusions [2]. Malocclusions are one of the major oral health problems ranking third after dental caries and periodontal disease [3]. It affects periodontal health and increases the risk of dental caries, traumatic dental injuries and temporomandibular joint problems [4]. Genetic, environmental, or a combination of both factors along with various local factors such as adverse or deleterious oral habits can cause malocclusions [5].

Malocclusions like various other dental disorders cause a profound impact on aesthetics and psychosocial behaviour of adolescents, thus affecting their self-esteem [6]. As a child grows, there is increased concern for dental appearance; therefore, dental appearance that is not acceptable to society tends to have an effect on a child's self-esteem and social interaction [3]. Therefore, an increase in interest in the relationship between malocclusions and OHRQoL has been observed among researchers and studies reported an association between malocclusions and poor OHRQoL [7, 8]. Malocclusions increase the negative impact on OHRQoL and therefore can in turn negatively affect general well-being of an individual. Simões et al. [9] reported that children with very severe malocclusions experienced greater negative impact on OHRQoL compared to those with mild or no malocclusions.

Global trends show an increase of aesthetic community awareness and treatment needs [7, 10] that needs to be addressed by Zambia's dental fraternity and health-care policymakers. Ghabrial et al. [11] studied the occlusal status of 9–12 years old Zambian children at 5 different urban schools and reported that 17 per cent of the participants required orthodontic treatment, of which, 5.2 per cent needed specialized treatment, while the magnitude and impact of malocclusions on OHRQoL in Zambian population is scarcely documented. Therefore, the current study aims at assessing the prevalence of malocclusions and its impact on OHRQoL among early adolescence school-going children in Ndola, Zambia.

## 2. Materials and Methods

### 2.1. Study Design and Participants

This is a cross-sectional study conducted over a period of eight weeks (March to May 2017) among children aged 12 to 14 years at four randomly selected public primary schools in Ndola district of Copperbelt Province located in central Zambia. A multistage cluster sampling technique was adopted. The list of primary schools was obtained from the District Education Board Secretary (DEBS) of Ndola district, which was grouped according to zones (zones 1 to 9), and there were a total of fifty-seven public primary schools. Four zones (1, 3, 6, and 9) out of nine in Ndola district were randomly selected, which accounted for twenty-five public primary schools, and subsequently, one school from each zone was randomly selected. Participants were selected using stratified random sampling method. Children under orthodontic or cosmetic dentistry treatment were excluded in this study. A pilot study was conducted where the prevalence of malocclusions was 49.9%, which was approximated to 50% and used to calculate the sample size. A total of 384 children aged 12 to 14 years were enrolled for the study after obtaining written consent from parents/guardians of participants. Participants were asked to complete a self-administered questionnaire, which included questions on sociodemographic details and Child Oral Health Impact Profile-Short Form 19 (COHIP-SF19) [12] and clinically examined for assessment of the presence of malocclusion using the Dental Aesthetic Index (DAI) [13, 14].

### 2.2. Compliance with Ethical Standards

Ethical clearance (Ref. number: TRC/C4/03/2017) was obtained from the Tropical Disease Research Centre (TDRC) Ethics Committee (Reg. number: 00002911; FWA: 00003729), Ndola, Zambia. Schools were approached through local educational authorities by obtaining permissions from the DEBS of Ndola and the Ministry of General Education, Zambia. Explanatory letters and consent forms were sent to parents a few days prior to the dental examination or interview, and only those children whose parents returned written consent forms were included. Written parental consent and each child's verbal consent were obtained from all the participants.

### 2.3. COHIP-SF19 Questionnaire

The COHIP-SF19 questionnaire, which is reliable and validated [12], was adopted. It consists of 19 items forming five conceptually distinct domains: Oral Health, Functional Well-Being, Social/Emotional Well-Being, School Environment, and Self-Image. Oral Health comprises specific oral health symptoms that are not necessarily related to one another (e.g., pain and spots on teeth). Functional Well-Being includes items pertaining to the child's ability to carry out specific everyday tasks or activities (e.g., speaking clearly and chewing). Social/Emotional Well-Being relates to peer interactions and mood states. School Environment incorporates items pertaining to tasks associated with the school environment. Self-Image addresses positive feelings about oneself. The statements in the COHIP-SF19 form were formatted to elicit self-reports from the children. Instructions for the items in the five domains were as follows: “Please read each statement carefully and choose the answer that best describes how you really feel in the past 3 months regarding your teeth, mouth, or face.”

### 2.4. Scoring of the COHIP-SF19 Questionnaire

Children rated whether they had “never = 0,” “almost never = 1,” “sometimes = 2,” “fairly often = 3,” and “almost all of the time = 4” experienced any of the situations listed in the past three months. Responses of the children were scored on a scale ranging from 0 (never) to 4 (almost all the time) with a higher score indicating poor OHRQoL. Scoring of the positively worded items was reversed, while scoring of the negatively worded items was not. The relations between OHRQoL and malocclusions were determined by considering the ratings “never = 0” and “almost never = 1” as “No impact on OHRQoL,” and “sometimes = 2,” “fairly often = 3,” and “almost all of the time = 4” as “Negative impact on OHRQoL.” So that, the minimum total COHIP-SF19 score is 0 and the maximum is 76. For the perception of impact on oral health quality, the total COHIP-SF19 scores between 0 and 19 were considered as “No impact on OHRQoL” and 20 to 76 were considered as “Negative impact on OHRQoL.” Thus, higher COHIP-SF19 scores reflect poor OHRQoL, while lower scores reflect good OHRQoL.

### 2.5. Clinical Examinations

Participants were examined in a classroom while lying on a bench facing the window in the direction of natural light. The DAI recommended by the World Health Organization (WHO) was used to assess dentofacial anomalies [13]. The index provides a link between clinical and aesthetic components mathematically and ends up with a single score. It also aims to predict clinical judgments of orthodontists by separating handicapping and nonhandicapping malocclusions. Ten occlusal characteristics were as follows: overjet, negative overjet, tooth loss, diastema, anterior open bite, anterior crowding, anterior diastema, width of the anterior irregularities (mandible and maxilla), and anterioposterior molar relationship that are related to dentofacial anomalies according to the three components of dentition, that is, spacing, crowding, and occlusion were used to assess the presence of malocclusion [13, 14]. The DAI score above 25 (DAI > 25) was considered as presence of malocclusion. Three calibrated examiners, namely, SAN (Specialist in Restorative Dentistry), RS (Specialist in Community and Preventive Dentistry), and ZK (Dental Therapist) did the clinical examination for the presence of malocclusion and the interexaminers' agreements were 0.91 (SAN and RS) and 0.89 (SAN and ZK). A group of eight fifth-year Bachelor of Dental Surgery (BDS) students of Michael Chilufya Sata School of Medicine (MCS SoM), Copperbelt University (CBU), assisted in the recording of data.

### 2.6. Statistical Analysis

All data collected were entered, cleaned, and analysed using SSPS software (version 20.0) for Windows (SPSS Inc., Chicago, Illinois, USA) to determine the levels and significance of quantitative data obtained in this study. Descriptive statistics such as frequency distribution and cross-tabulations were used to summarize the data. Bivariate analysis (chi-square analysis) was conducted in an attempt to describe and establish the relationship between sociodemographics (age and sex) and OHRQoL and also between sociodemographics and prevalence of malocclusions. A multivariate logistic regression analysis was conducted to ascertain the association between independent variables (sociodemographic and malocclusion) and OHRQoL (dependent variable) that were significant at bivariate analysis. Statistical significance was set at *p* < 0.05.

## 3. Results

Table 1 presents the frequency distribution of participants according to sociodemographic characteristics. A total of 384 school-going adolescence children participated in this study. Of the participants, 53.6% were female, 41.7% were aged 13 years, and 43.5% were from grade six.

Table 2 summarizes the frequency distribution of participants' response to COHIP-SF19 components. The COHIP-SF19 scores ranged from 0 to 54, mean ± SD = 10.59 ±8.2. According to COHIP-SF19 scores, 25.6% of the children had experienced oral health problems in the previous three months from the date of interview. Of the Oral Health Well-Being components of COHIP-SF19, bad breath (40.4%) and bleeding gums (39.9%) were most reported. The functional problems were experienced by 13.8% of the children, and of its components, difficulty in keeping teeth clean (47.4%) was most reported. The socioemotional impacts were experienced by 5.5% of the children, and of its components, avoided smiling or laughing with other children (10.4%) was most reported. Impact on school or environmental domain was reported by 2.9%, and in particular, 7% of children did not want to speak/read out loud in class. Whereas impact on self-image domain accounted for 0.5%, and in particular, 1.3% of children felt that they were not attractive.

Table 3 shows the relationship between OHRQoL and sociodemographic variables of participants. According to the total COHIP-SF19 dichotomised scores of participants, the overall reported impact on OHRQoL was 11.7%. The eldest age group (children aged 14 years) reported the highest oral health-related impact profile (18.6%) when compared to other age groups and the least impact value (1.1%) reported by the children aged 12 years. There was a statistically highly significant relationship (*p* < 0.001) between age and OHRQoL among the children. In relation to sex and OHRQoL, the statistically significant relationship (*p* < 0.001) was found among the children, and moreover, the female participants reported the higher impact on OHRQoL (18.0%) than their male counterparts (4.5%).

Table 4 shows the prevalence of malocclusions according to age and sex of participants. The overall prevalence of malocclusions was 27.9%. A greater proportion of males (33.7%) were affected by malocclusions than females (22.8%). The prevalence of malocclusions was statistically significant in relation to sex (*p*=0.018), but not significant in relation to age (*p*=0.987).

Table 5 summarizes the relationship between OHRQoL and different kinds of malocclusions. The most prevalent malocclusion was spacing (10.9%) followed by diastema (9.9%) and crowding (7.6%), and the least prevalent was missing teeth in the maxilla (0.5%). The impact of overall malocclusions on OHRQoL was 29.9% as compared to no malocclusions (4.7%) and showed the statistically significant relationship (*p* < 0.001). Out of eight reported malocclusions in the study, anteriomaxillary overjet showed highest (100%) impact and statistically significant association (*p* < 0.001) with OHRQoL compared to the children without anteriomaxillary overjet. Anterioposterior molar relation (*p*=0.027), anteriomandibular overjet (*p*=0.010), spacing (*p* < 0.001), crowding (*p*=0.001), and diastema (*p* < 0.001) also showed the statistically significant impact on OHRQoL of children as compared to the children with no respective malocclusions. No impact was recorded on OHRQoL in children with missing teeth in the maxilla (0%). No statistically significant impact on OHRQoL was observed in children with missing teeth in the maxilla (*p*=0.605) and children with vertical anterior open bite (*p*=0.123) as compared to the children with no respective malocclusions.

A multivariate logistic regression analysis was performed to assess the influence of sociodemographics and malocclusions on OHRQoL (Table 6). All significant variables in bivariate analysis were included except anteriomaxillary overjet and missing teeth in the maxilla since their odds ratio was undefined due to the presence of null values. The model 4 of multivariate logistic regression analysis revealed that age, sex, spacing, crowding, and diastema were independently associated with OHRQoL. The impact on OHRQoL increased by an increase in age. The children aged 13 (AOR: 19.02, 95% CI (2.06–175.85)) and 14 years (AOR: 27.34, 95% CI (2.97–251.36)) were 19 and 27 times more likely to report higher impact on OHRQoL, respectively, than children aged 12 years. Females were 7 times (AOR: 7.40, 95% CI (2.78–19.67)) more likely to report higher impact on OHRQoL than males. The children with crowding (AOR: 3.93, 95% CI (1.46–10.60)), diastema (AOR: 3.96, 95% CI (1.31–11.97)), and spacing (AOR: 4.32, 95% CI (1.38–13.55)) were around four times more likely to report higher impact on OHRQoL than the children without respective malocclusions.

## 4. Discussion

This is the first study in Zambia, which looked at the prevalence of malocclusions and their impact on OHRQoL among adolescence school-going children. Our findings on OHRQoL revealed that oral health problems (domain 1) were highly affected among five domains in COHIP-SF19, and in which, bad breath and bleeding gums were the most reported components. Functional problems (domain 2) were second most affected, and in which, difficulty in keeping teeth clean was a most reported item among 19 items in COHIP-SF19. Whereas the least reported domain was Self-Image. In contrary to the findings of this study, researchers reported that the effects of malocclusions are mainly on the Social/Emotional Well-Being [8, 15] and Self-Image [16]. Castro et al. [17] reported that eating and cleaning mouth, which are the components of Functional Well-Being domain in COHIP-SF19, had the highest impact on OHRQoL, and it supports the findings of this study on “difficulty in keeping teeth clean” but contrary to the findings on “difficulty in eating foods.”

The impact on OHRQoL is usually expected to be greatly significant in females than males, since males may be less self-conscious about their appearance [18]. The findings of this study also showed significant interference (*p* < 0.001) in females as compared to males, which is in concordance with the study done by Scapini et al. [15] and Asokan et al. [16]. Children between the age of 12 and 14 were most likely to have an impact of malocclusions on OHRQoL, and it may be due to increasing centrality of peer crowd and clique dynamics in children's lives and their preoccupation with others' views of self [19]. The study on the association between OHRQoL and age revealed that children aged 14 years recorded higher COHIP-SF19 score than other lower age groups. The older the children get, the more their malocclusions affect their OHRQoL [20], which is reflected in the study findings.

The overall prevalence of malocclusions among the study group was slightly lower than that reported for children in Brazil [21], Tanzania [22], India [23], and Mongolia [24]. This might probably be due to ethnic differences among the comparative groups. The gender-wise analysis revealed that the prevalence of malocclusions was higher in male children as compared to female children. The study further established that there was a significant (*p*=0.005) relationship between the gender and prevalence of malocclusions among the children, which confirmed the existence of an association between the gender and prevalence of malocclusions. Earlier studies carried out in Kenya [25] and Iran [26] also reported that more males were found to have malocclusion than females, although the difference was not significant. This finding is inconsistent with other studies carried out in Nigeria where gender differences were not found [27, 28].

Our findings revealed that spacing, diastema, and crowding were the most prevalent malocclusions, and the least prevalent was missing teeth in the maxilla. Similar to our findings, Shivakumar et al. [29] and Ajayi [27] reported crowding and spacing as most prevalent malocclusions among school children, and the latter one further stated that spacing of the upper anterior segment was more common than crowding in Nigerian school children. Anosike et al. [30] reported that spacing was mostly recorded while crowding was most prevalent and missing teeth was less prevalent in Nigerian school children, which also supports our findings. Higher prevalence of diastema in our study may be due to racial factor. According to Lavelle's [31] report, the prevalence of the maxillary median diastema was greater in Africans (West Africa) than in Caucasians (British) or Mongoloids (Chinese from Hong Kong and Malaya). Horowitz [32] reported that black children exhibit a higher prevalence of midline diastema than do white children.

Malocclusions are associated with impaired OHRQoL and risk of personal dissatisfaction with visible malocclusions and therefore considered as an important treatment-motivating factor [8]. The study on OHRQoL in relation to malocclusions revealed that different types of malocclusions had a different level of impact on OHRQoL and hence different level of negative association with OHRQoL of participants. The findings of this study showed that there was a significant association (*p* < 0.001) between overall malocclusions and its impact on OHRQoL. Klages et al. [33] reported that even minor differences in dental aesthetics of an individual might have a significant effect on perceived OHRQoL. In the present study, anteriomaxillary overjet, anterioposterior molar relation, anteriomandibular overjet, spacing, crowding, and diastema showed significant negative association with OHRQoL. A multivariate logistic regression analysis revealed that age, sex, spacing, crowding, and diastema showed significant impact on OHRQoL. The strength of impact was increasing with an increase in age and higher in females than males. Spacing, crowding, and diastema showed clear inverse association with OHRQoL of participants. Anosike et al. [30] reported that diastema, missing teeth, and maxillary and mandibular irregularities were significantly associated with OHRQoL in Nigerian school-going children, which partially supports our findings. Likewise, a systematic review, which included 22 research reports, also supports with its report that increased overjet, crowding, and diastema had been associated with bullying and a lower self-esteem among teenagers and showed negative effects on OHRQoL in children and adolescents [8]. Johal et al. [34] reported that an increased overjet and a spaced dentition had a significant negative impact on OHRQoL, which is in agreement with the findings of this study.

The findings of the study highlighted the magnitude and impact of malocclusions on OHRQoL of early adolescents in Ndola district of Copperbelt Province, Zambia. The study findings also emphasize the importance of considering malocclusions as one among the major oral health problems in Ndola, Zambia. Therefore, the assessment of malocclusions and measurement of OHRQoL should be an essential component of oral health screening since it has a significant negative impact on OHRQoL as evident from the study. A few limitations must be considered when interpreting our findings: first, lack of external validity, which is needed to generalize our results to whole Zambian adolescents; second, the absence of evaluation of dental caries and periodontal diseases in the study, which may also influence participants' OHRQoL. Thus, further large-scale study with various age and ethnic groups including evaluation of dental caries and periodontal diseases along with malocclusions may be needed to address the OHRQoL of the Zambian population.

## 5. Conclusion

The study estimated the prevalence of malocclusion and established their negative association with OHRQoL among early adolescents in Ndola, Zambia. Age, sex, spacing, crowding, and diastema were significantly associated with the higher impact on OHRQoL. Though the prevalence of malocclusion was significantly higher in males, significant impact on OHRQoL was found in females rather than their male counterparts. The findings of the study further showed that increase in age and being a female appear to be strong influential factors on children's perception of OHRQoL. Further large-scale studies with different age and ethnic groups are needed to establish an inference on association between malocclusions and OHRQoL of Zambian adolescents.

## Figures and Tables

**Table 1:** Frequency distribution of participants by sociodemographic characteristics (*n*=384).

Participants	Sex		Age (years)			Grade of study			School			
	Male	Female	12	13	14	6th	7th	8th	1	2	3	4
*n*	178	206	95	160	129	167	96	121	81	95	108	100
%	46.4	53.6	24.7	41.7	33.6	43.5	25.0	31.5	21.1	24.7	28.1	26.0

*n*: number; %: percentage.

**Table 2:** Frequency distribution (*n* (%)) of the responses for COHIP-SF19 items (*n*=384).

COHIP-SF19 items	Almost all the time	Fairly often	Sometimes	Almost never	Never
Domain 1: Oral Health Well-Being					
Q1: had pain in your teeth/toothache	2 (0.5)	13 (3.4)	7 (1.8)	94 (24.5)	268 (69.8)
Q2: had discoloured teeth or spots on your teeth	6 (1.6)	16 (4.2)	55 (14.3)	68 (17.7)	239 (62.2)
Q3: had crooked teeth or spaces between your teeth	4 (1.0)	12 (3.1)	14 (3.6)	81 (21.1)	273 (71.1)
Q4: had bad breath	1 (0.3)	2 (0.5)	152 (39.6)	92 (24.0)	137 (35.7)
Q5: had bleeding gums	3 (0.8)	8 (2.1)	142 (37.0)	49 (12.8)	182 (47.4)
Domain 1 subtotal	1 (0.3)	13 (3.4)	84 (21.9)	173 (45.0)	113 (29.4)

Domain 2: Functional Well-Being					
Q6: had difficulty eating foods you would like to eat	3 (0.8)	6 (1.6)	10 (2.6)	105 (27.3)	260 (67.7)
Q7: had trouble sleeping	2 (0.5)	12 (3.1)	28 (7.3)	69 (18.0)	273 (71.1)
Q8: had difficultly saying certain words	2 (0.5)	7 (1.8)	16 (4.2)	72 (18.8)	287 (74.7)
Q9: had difficulty keeping your teeth clean	21 (5.5)	45 (11.7)	116 (30.2)	69 (18.0)	133 (34.6)
Domain 2 subtotal	0 (0)	12 (3.1)	41 (10.7)	216 (56.3)	115 (29.9)

Domain 3: Social/Emotional Well-Being					
Q10: been unhappy or sad	1 (0.3)	2 (0.5)	5 (1.3)	62 (16.1)	314 (81.8)
Q11: felt worried or anxious	1 (0.3)	2 (0.5)	6 (1.6)	94 (24.5)	281 (73.2)
Q12: avoided smiling or laughing with other children	8 (2.1)	12 (3.1)	20 (5.2)	76 (19.8)	268 (69.8)
Q13: felt that you look different	3 (0.8)	8 (2.1)	12 (3.1)	65 (16.9)	296 (77.1)
Q14: been worried about what other people think about you	4 (1.0)	9 (2.3)	7 (1.8)	73 (19.0)	291 (75.8)
Q15: been teased, bullied, or called names by other children	5 (1.3)	6 (1.6)	13 (3.4)	51 (13.3)	309 (80.5)
Domain 3 subtotal	1 (0.3)	5 (1.3)	15 (3.9)	136 (35.4)	227 (59.1)

Domain 4: School Environment					
Q16: missed school for any reason	0 (0.0)	1 (0.3)	2 (0.5)	48 (12.5)	333 (86.7)
Q17: not wanted to speak/read out loud in class	2 (0.5)	7 (1.8)	18 (4.7)	30 (7.8)	327 (85.2)
Domain 4 subtotal	0 (0)	1 (0.3)	10 (2.6)	63 (16.4)	310 (80.7)

Domain 5: Self-Image					
Q18: been confident	222 (57.8)	46 (12.0)	114 (29.7)	2 (0.5)	0 (0.0)
Q19: felt that you were attractive	47 (12.2)	116 (30.2)	216 (56.3)	3 (0.8)	2 (0.5)
Domain 5 subtotal	24 (6.3)	225 (58.6)	133 (34.6)	2 (0.5)	0 (0)

*n*: number; %: percentage; COHIP-SF19: Child Oral Health Impact Profile-Short Form 19.

**Table 3:** Relationship between OHRQoL and sociodemographic variables.

Sociodemographics	Groups	Number of pupils	Impact on OHRQoL				*χ* ^2^	*p* value
			No		Yes			
			*n*	%	*n*	%		
Age (years)	12	95	94	98.9	1	1.1	16.454	<0.001^*∗*^
	13	160	140	87.5	20	12.5	—	—
	14	129	105	81.4	24	18.6	—	—

Sex	Male	178	170	95.5	8	4.5	16.739	<0.001^*∗*^
	Female	206	169	82.0	37	18.0	—	—

Overall	—	384	339	88.3	45	11.7	—	—

*n*: number; %: percentage; OHRQoL: oral health-related quality of life; ^*∗*^*p* < 0.05 is significant.

**Table 4:** Prevalence of malocclusions according to sociodemographics of participants.

Sociodemographics	Groups	Number of pupils	Malocclusions				*χ* ^2^	*p* value
			Absence		Presence			
			*n*	%	*n*	%		
Age (years)	12	95	68	71.6	27	28.4	0.025	0.987
	13	160	116	72.5	44	27.5	—	—
	14	129	93	72.1	36	27.9	—	—

Sex	Male	178	118	66.3	60	33.7	5.636	0.018^*∗*^
	Female	206	159	77.2	47	22.8	—	—

Overall	—	384	277	72.1	107	27.9	—	—

*n*: number; %: percentage; ^*∗*^*p* < 0.05 is significant.

**Table 5:** Relationship between OHRQoL and malocclusions.

Malocclusions	Occurrence	Impact on OHRQoL				Total		*χ* ^2^	*p* value
		No		Yes					
		*n*	%	*n*	%	*n*	%		
Anteriomaxillary overjet	No	339	90.2	37	9.8	376	97.9	61.549	<0.001^*∗*^
	Yes	0	0.0	8	100	8	2.1	—	—

Anterioposterior molar relation	No	332	89.2	39	10.8	361	94.0	4.882	0.027^*∗*^
	Yes	17	73.9	6	26.1	23	6.0	—	—

Vertical anterior open bite	No	321	88.9	40	11.1	361	94.0	2.374	0.123
	Yes	18	78.3	5	21.7	23	6.0	—	—

Anteriomandibular overjet	No	337	88.7	43	11.3	380	99.0	5.726	0.010^*∗*^
	Yes	2	50	2	50	4	1.0	—	—

Missing teeth in maxilla	No	337	88.2	45	11.8	382	99.5	0.267	0.605
	Yes	2	100	0	0.0	2	0.5	—	—

Spacing	No	310	90.6	32	9.4	342	89.1	16.863	<0.001^*∗*^
	Yes	29	69.0	13	31.0	42	10.9	—	—

Crowding	No	319	89.9	36	10.1	355	92.4	11.313	0.001^*∗*^
	Yes	20	69.0	9	31.0	29	7.6	—	—

Diastema	No	313	90.5	33	9.5	346	90.1	16.079	<0.001^*∗*^
	Yes	26	68.4	12	31.6	38	9.9	—	—

Overall malocclusions	No	264	95.3	13	4.7	277	72.1	47.429	<0.001^*∗*^
	Yes	75	70.1	32	29.9	107	27.9	—	—

*n*: number; %: percentage; OHRQoL: oral health-related quality of life; ^*∗*^*p* < 0.05 is significant.

**Table 6:** Influence of sociodemographics and malocclusions on OHRQoL: multivariate logistic regression (AOR).

Variables	Categories	Model 1	Model 2	Model 3	Model 4
		AOR (95% CI)	AOR (95% CI)	AOR (95% CI)	AOR (95% CI)
Age	12	1	1	1	1
	13	18.64^*∗∗∗*^ (2.05–169.83)	19.18^*∗∗∗*^ (2.09–176.07)	18.85^*∗∗∗*^ (2.08–170.71)	19.02^*∗∗∗*^ (2.06–175.85)
	14	25.01^*∗∗∗*^ (2.76–226.47)	25.58^*∗∗∗*^ (2.80–233.76)	25.22^*∗∗∗*^ (2.79–227.97)	27.34^*∗∗∗*^ (2.97–251.36)

Sex	Male	1	1	1	1
	Female	7.90^*∗∗∗*^ (2.87–21.78)	7.67^*∗∗∗*^ (2.82–20.86)	7.56^*∗∗∗*^ (2.82–20.31)	7.40^*∗∗∗*^ (2.78–19.67)

Crowding	No	1	1	1	1
	Yes	3.05^*∗∗*^ (1.04–8.96)	3.19^*∗∗*^ (1.10–9.22)	3.16^*∗∗*^ (1.10–9.07)	3.93^*∗∗∗*^ (1.46–10.60)

Spacing	No	1	1	1	1
	Yes	4.37^*∗∗*^ (1.35–14.10)	4.55^*∗∗*^ (1.42–14.55)	4.62^*∗∗∗*^ (1.47–14.55)	4.32^*∗∗*^ (1.38–13.55)

Diastema	No	1	1	1	1
	Yes	4.17^*∗∗*^ (1.32–13.20)	4.15^*∗∗*^ (1.30–13.21)	4.00^*∗∗*^ (1.33–12.00)	3.96^*∗∗*^ (1.31–11.97)

Anteriomandibular overjet	No	1	1	1	—
	Yes	10.34 (0.32–331.20)	13.32 (0.49–363.72)	12.45 (0.46–337.92)	—

Vertical anterior open bite	No	1	1	—	—
	Yes	0.84 (0.20–3.56)	0.87 (0.21–3.64)	—	—

Anterioposterior molar relation	No	1	—	—	—
	Yes	1.36 (0.35–5.23)	—	—	—

Total	—	384	384	384	384

AOR: adjusted odds ratio; CI: confidence interval; OHRQoL: oral health-related quality of life; ^*∗∗∗*^*p* < 0.01; ^*∗∗*^*p* < 0.05; ^*∗*^*p* < 0.1 (*p* < 0.05 is significant).

## Data Availability

All data generated or analysed during this study are included in this article.

## Abbreviations

OHRQoL: Oral health-related quality of life
COHIP-SF19: Child Oral Health Impact Profile-Short Form 19
DAI: Dental Aesthetic Index.
